# Optimised passive marker device visibility and automatic marker detection for 3-T MRI-guided endovascular interventions: a pulsatile flow phantom study

**DOI:** 10.1186/s41747-022-00262-4

**Published:** 2022-02-24

**Authors:** Han Nijsink, Christiaan G. Overduin, Patrick Brand, Sytse F. De Jong, Paul J. A. Borm, Michiel C. Warlé, Jurgen J. Fütterer

**Affiliations:** 1 Department of Medical Imaging, Radboudumc, Geert Grooteplein Zuid 10, 6525 Nijmegen, GA The Netherlands; 2 Department of Cardiothoracic Surgery, Radboudumc, Nijmegen, The Netherlands; 3 Nano4Imaging GmbH, Düsseldorf, Germany; 4 Department of Vascular and Transplant Surgery, Radboudumc, Nijmegen, The Netherlands

**Keywords:** Deep learning, Endovascular procedures, Magnetic resonance imaging, Phantom studies, Pulsatile flow

## Abstract

**Background:**

Passive paramagnetic markers on magnetic resonance imaging (MRI)-compatible endovascular devices induce susceptibility artifacts, enabling MRI-visibility and real-time MRI-guidance. Optimised visibility is crucial for automatic detection and device tracking but depends on MRI technical parameters and marker characteristics. We assessed marker visibility and automatic detection robustness for varying MRI parameters and marker characteristics in a pulsatile flow phantom.

**Methods:**

Guidewires with varying iron(II,III) oxide nanoparticle (IONP) concentration markers were imaged using gradient-echo (GRE) and balanced steady-state free precession (bSSFP) sequences at 3 T. Furthermore, echo time (TE), slice thickness (ST) and phase encoding direction (PED) were varied. Artifact width was measured and contrast-to-noise ratios were calculated. Marker visibility and image quality were scored by two MRI interventional radiologists. Additionally, a deep learning model for automatic marker detection was trained and the effects of the parameters on detection performance were evaluated. Two-tailed Wilcoxon signed-rank tests were used (significance level, *p* < 0.05).

**Results:**

Medan artifact width (IQR) was larger in bSSFP compared to GRE images (12.7 mm (11.0–15.2) *versus* 8.4 mm (6.5–11.0)) (*p* < 0.001) and showed a positive relation with TE and IONP concentration. Switching PED and doubling ST had limited effect on artifact width. Image quality assessment scores were higher for GRE compared to bSSFP images. The deep learning model automatically detected the markers. However, the model performance was reduced after adjusting PED, TE, and IONP concentration.

**Conclusion:**

Marker visibility was sufficient and a large range of artifact sizes was generated by adjusting TE and IONP concentration. Deep learning-based marker detection was feasible but performance decreased for altered MR parameters. These factors should be considered to optimise device visibility and ensure reliable automatic marker detectability in MRI-guided endovascular interventions.

## Key points

- Visibility and detectability of passive marker endovascular devices for MRI-guidance were investigated.
- Passive marker size, visibility, and detectability can be tailored during MRI-guided interventions.
- Deep learning-based automatic passive marker detection showed promising results.

## Background

Endovascular interventions have been embraced by interventional radiologists and vascular surgeons to reduce the surgical impact related to open surgery. At the same time, many other disciplines introduced endovascular procedures with applications in paediatrics [1], cardiology [2, 3], neurology [4], and oncology [5, 6].

Currently, endovascular interventions are mainly guided by fluoroscopy, but this technique is hampered by limited two-dimensional visualisation, use of contrast agent and radiation burden to patient and physician [7, 8]. MRI-guidance offers some potential advantages over fluoroscopy-guidance, relating to its intrinsic soft tissue contrast, and the possibility of functional imaging, without the need for iodinated contrast agents or ionising radiation [9]. The last advantage is of particular concern in paediatric and young adult patients due to the cumulative stochastic effects of radiation exposure. Although literature describes feasibility of MRI-guided endovascular interventions in humans, these studies have been published before 2006 [10–12]. Clinical adoption of MRI-guided vascular interventions is likely hampered by main challenges such as optimising between decreased image contrast and in plane resolution and higher frame rates (> 1 frames/s), MRI safety, costs and limited patient access. In addition, there is still a limited availability of MRI conditional and CE marked endovascular devices [7–9].

MRI-guided endovascular interventions require MRI-compatible, and visible devices such as guidewires and catheters. Visibility of those devices is usually realised by enhancement with active, semiactive or passive markers [13]. Passive paramagnetic markers induce susceptibility artifacts, are inherently safe, straightforward and do not require additional imaging or tracking prerequisites [14, 15]. For easy and automatic detection, marker-induced artifacts should be clearly and separately visible whilst not impeding local anatomical detail [9, 16, 17]. Consequently, the desired artifact size may vary for different intervention types. For example, guidance of vascular devices through the aorta and the left ventricle can be achieved by using large artifacts. Contrary, guidance through complex vascular structures or accurate positioning in complex anatomical regions requires smaller artifacts to pertain sufficient anatomical detail [16, 18].

Optimising marker visibility is crucial in improving MRI-guided endovascular interventions and knowledge of the effects of different parameters on artifact size and appearance is therefore important in designing and using endovascular devices. In addition, device tracking allows continuous visualisation of the device and is important for improving the workflow. For passive markers, the performance of automatic detection relies on the marker appearance and visibility.

Based on static phantoms without flow, it is known that passive marker artifact size is positively related to main magnetic field strength [19–24], echo time (TE) [20–22, 24, 25] and the amount and type of paramagnetic material [23–25]. It is, however, unknown how artifacts are influenced under pulsatile blood flow in the vascular system. Furthermore, most research focused on 1.5-T MRI systems since these are generally accepted for cardiac MRI-guided interventions [3, 9]. MRI systems with a higher main magnetic field intrinsically provide a higher signal-to-noise ratio which can potentially be used to increase the temporal resolution during interventions or provide improved functional imaging capability [21]. Therefore, the first aim of this study is to quantify the effects of altering MRI parameters, sequence type and paramagnetic material load on passive marker artifact visibility in a pulsatile flow phantom at 3 T. Secondly, we evaluated robustness of deep learning based automatic marker detection to variations in those MRI and marker parameters.

## Methods

### Phantom and guidewires

**Fig. 1:**
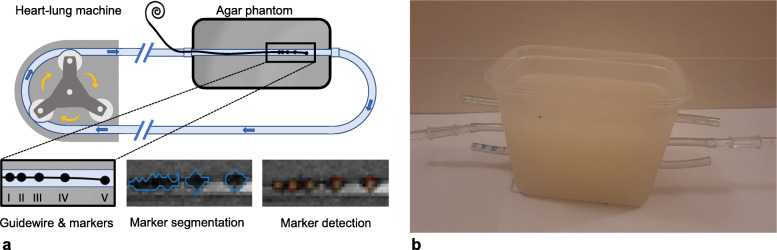
**a** Schematic drawing of the guidewire with markers inserted in the flow phantom. Examples of segmented markers and automatically detected markers are visualised. **b** The phantom used for the experiments

A vascular flow phantom was designed by placing an 8-mm tube in a soft tissue mimicking agar solution (Sigma-Aldrich, St. Louis, Missouri, USA) and connecting the tube to a heart-lung machine (HL 20, Getinge, Gothenburg, Sweden) (Fig. 1). The size of the tube was selected to resemble an average peripheral artery diameter. To mimic human blood flow, a blood-mimicking fluid (water with 3% glycerol and 0.15 ml/L of gadoterate meglumine (Dotarem, Guerbet, Princeton, New Jersey) [26] was pumped through the tube with a pulsatile flow of 0.4 L/min at 60 beats per minute. In this study, the markers of the EmeryGlide® guidewire (Nano4Imaging GmbH, Düsseldorf, Germany) with a diameter of 0.035 inch were replaced by five markers. These markers were identical, cylindrical markers of equal concentration with a length of 1.5 mm and a volume of 0.036 μL. Markers were placed with different intermarker distances (20, 15, 10, and 5 mm), with the first marker being located at the exact tip. A total of four different guidewires with different iron(II,III) oxide (Fe_3_O_4_) nanoparticle (IONP) concentrations for each wire (6.25, 12.5, 25, and 50 mg/mL) were used. These concentrations are lower than those currently used in the CE marked EmeryGlide® (100 mg/mL), and were selected since preliminary experiments revealed that the concentration of 10 mg/mL resulted in relatively large (> 20 mm) marker artifacts.

### MRI protocol

The phantom was placed in the centre of the bore, with the tube (and thereby the flow direction) and guidewire oriented along the main magnetic field (B_0_), approximating the orientation of the aorta. Although parts of the aorta or smaller peripheral arteries may run at oblique angles to B_0_, it is known that for small passive markers this has negligible effect on artifact size and appearance [27]. Image acquisition was performed with a 3-T clinical MRI system (Magnetom Skyra, Siemens, Erlangen, Germany) with an anteriorly placed 18-channel phased array body coil and integrated spine matrix coil.

Two baseline sequences that are commonly used to guide interventional endovascular procedures were selected: (i) a two-dimensional spoiled gradient recalled echo (GRE) sequence with following technical parameters: echo time (TE) / repetition time (TR) 2.48/4.6 ms; frame rate 3.3 frames/s; matrix size 144 × 144; flip angle 12°; pixel size 1.74 × 1.7 mm; slice orientation sagittal; phase encoding direction (PED) perpendicular to B_0_ (anterior-posterior direction); slice thickness (ST) 5 mm); (ii) a two-dimensional balanced steady-state free precession (bSSFP) sequence with the same parameters used of the GRE sequence, except for TE/TR 1.47/2.9 ms, flip angle 39°, and frame rate (5 frames/s). Prescan normalisation, parallel imaging GRAPPA acceleration (factor 2) and partial Fourier reconstruction (sampling factor 6/8) were applied in both sequences.

The guidewires with the four different IONP concentration markers were consecutively scanned using both baseline sequences. Afterwards, the guidewire with the 6.25 mg/mL IONP concentration markers was scanned by varying single technical parameters from the baseline sequences. The varied MRI parameters were TE, PED, and ST (Table 1). All possible combinations of different TE and marker concentrations were adjusted to evaluate the combined influence on artifact size.

**Table 1:** Evaluated parameters with corresponding baseline and varied values

Evaluated parameters	Baseline value	Varied values		
IO concentration (mg/mL)	6.25	12.5	25	50
GRE, TE/TR (ms)	2.48/4*.*6 (a)	2.54/4.8 (b)	3.05/5.8 (c)	6.49/12.1 (d)
bSSFP, TE / TR (ms)	1.47/2.9 (e)	1.54/3.1 (f)	1.96/3.9 8 (g)	4.85/9.6 (h)
Slice thickness (mm)	5		10	
Phase encoding direction	Perpendicular		Parallel	

*bSSFP* Balanced steady-state free precession, *GRE* Gradient echo, *IO* Iron(II,III) oxide, *TE* Echo time, *TR* Repetition time. Bandwidth for a–d sequences was 1,445, 990, 505, 130 Hz/pixel, respectively. Bandwidth for e–h sequences was 1,510, 990, 505, 130 Hz/pixel, respectively

For each parameter, 30 consecutive images were acquired. Furthermore, reference images of the phantom without guidewire were acquired for each setting. Slice orientation, slice position (through the centre of the tube), phantom location and flow parameters remained unchanged for all tested parameters.

### Image analysis

Marker visibility was quantitatively analysed by measuring artifact size and contrast-to-noise ratio (CNR) and qualitatively through image quality assessment by two experienced MRI interventional radiologists (J.F. and K.O. with more than 15 and 9 years of experience, respectively).

#### Quantitative analysis

**Fig. 2:**
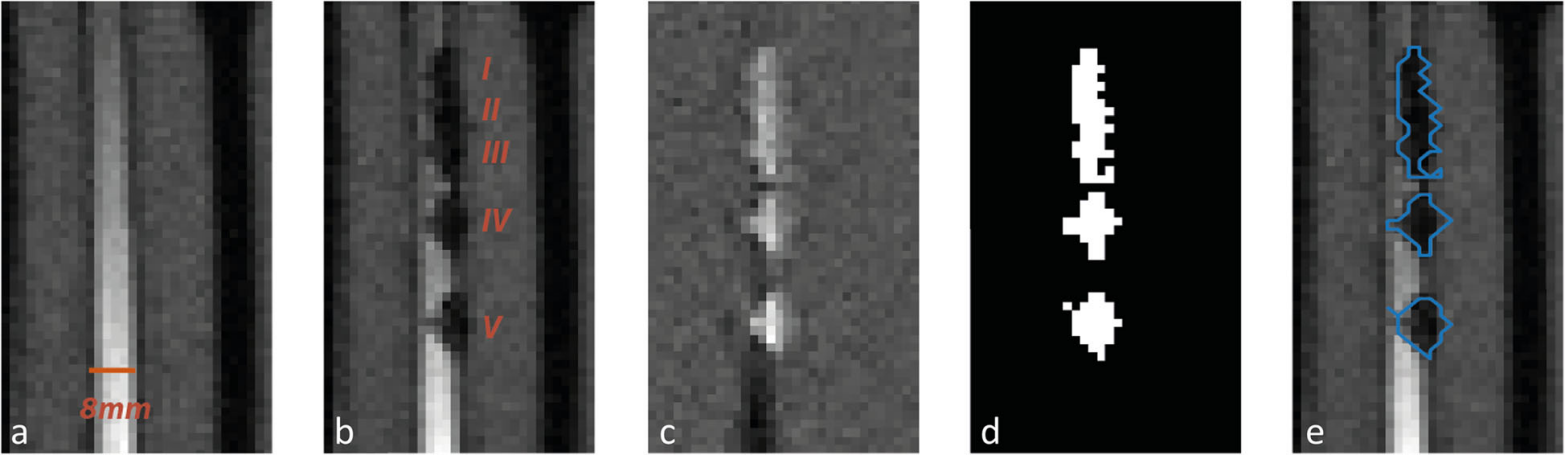
Segmentation of the artifact from a sagittal gradient echo image with 6.25 mg/mL iron(II,III)oxide markers (echo time 2.48 ms, slice thickness 5 mm, phase encoding direction: parallel). **a** Reference image of the 8-mm vessel without guidewire. **b** Marker image with inserted guidewire. **c** Result after subtraction of marker image from reference image. **d** Mask image with segmented markers used to calculate mean artifact width. **e** Outline of segmented markers overlaid on the marker image

All images were imported in MATLAB (R2018b, MathWorks Inc., Natick, MA, USA) to calculate artifact size and CNR. The first five images of each dataset were discarded to mitigate influence of saturation effects. Because the pulsatile flow introduced fluctuating signal intensities, reference images and images with guidewires were paired using the blood signal intensity. Thereafter, automatic segmentation of the image artifact was performed for the remaining 25 images in each dataset by subtracting the marker image from the paired reference image. The resulting subtraction image was used to calculate a standardised threshold (mean + 3 times the standard deviation of the agar signal intensity). Voxels within the subtraction image with an intensity higher than the threshold were assigned to a marker artifact segmentation mask (Fig. 2). Artifact size was specified as the mean artifact width, calculated by averaging the width of the artifact mask perpendicular to the guidewire. Average blood-, marker artifact-, and surrounding agar intensity values in the original images were used to calculate CNR between marker artifact and blood (blood-artifact CNR) and CNR between marker artifact and agar (agar-artifact CNR) [28]. The regions of interest for calculating the signal of blood, agar and noise were manually selected and unchanged for all images within the different datasets. The size of the regions of interest of blood, agar and noise were 2 × 17, 6 × 54 and 31 × 141 voxels, respectively. Images with artifacts unrelated to markers overshadowing the region of interest were eliminated in the quantitative analysis.

#### Qualitative analysis

Datasets were randomly presented in Osirix (Pixmeo, Geneva, Switzerland). The two MRI interventional radiologists independently scored the images for marker visibility, overall image quality, and usefulness for guidance of peripheral vascular interventions using a five-point Likert scale as unacceptable (1), poor (2), acceptable (3), good (4), or excellent (5). Observers scored the image quality by taking into account the contrast between markers, flowing blood and surrounding soft tissue and had to decide whether the image quality of all structures was sufficient to guide the procedure within the phantom setup. This analysis was used to quantify the subjective difference in image appearance between the different MRI and marker parameters in an objective manner.

#### Automatic marker detection

A U-Net [29] deep learning model was trained to detect markers on GRE images by predicting marker location likelihood maps. The labels for this training task were constructed as follows. Firstly, marker centre locations were manually annotated by one of the authors (H.N.). Secondly, a likelihood map was produced for each marker by placing a two-dimensional Gaussian kernel at the corresponding marker centre and variance, σ = (1,1). Thirdly, the label was obtained by combining all these marker likelihood maps into one map by taking the element-wise mean of all maps and normalising its values in the range [0,1]. The dataset consisted of a trainset of 30 baseline GRE images and labels, and a validation set of 10 baseline GRE images and labels. The model was trained for 400 epochs with a mean squared error loss and the Adam optimiser [30], learning rate = 1e-4. Random rotation, translation, scale and crop data augmentation techniques were used during training. The model was evaluated on 10 baseline GRE images and on 25 GRE images for the parameter configurations. To evaluate the detection performance, several steps were executed. Firstly, local maxima in the likelihood map were extracted using MATLAB’s local maxima function, using a pixel connectivity of 8. Secondly, true positive and false positive detections were calculated for a range of likelihood thresholds (0.1–0.9 with 0.05 steps). If the detected marker location deviated not more than 2 voxels from the annotated marker location, the marker was specified as correctly detected.

### Statistical analysis

Statistical analysis was performed using MATLAB. Artifact width and CNRs were compared between groups using the two-tailed Wilcoxon signed rank test. Image quality assessment scores were combined and the median scores of both observers were displayed. *p*-values < 0.05 were considered statistically significant.

## Results

**Fig. 3:**
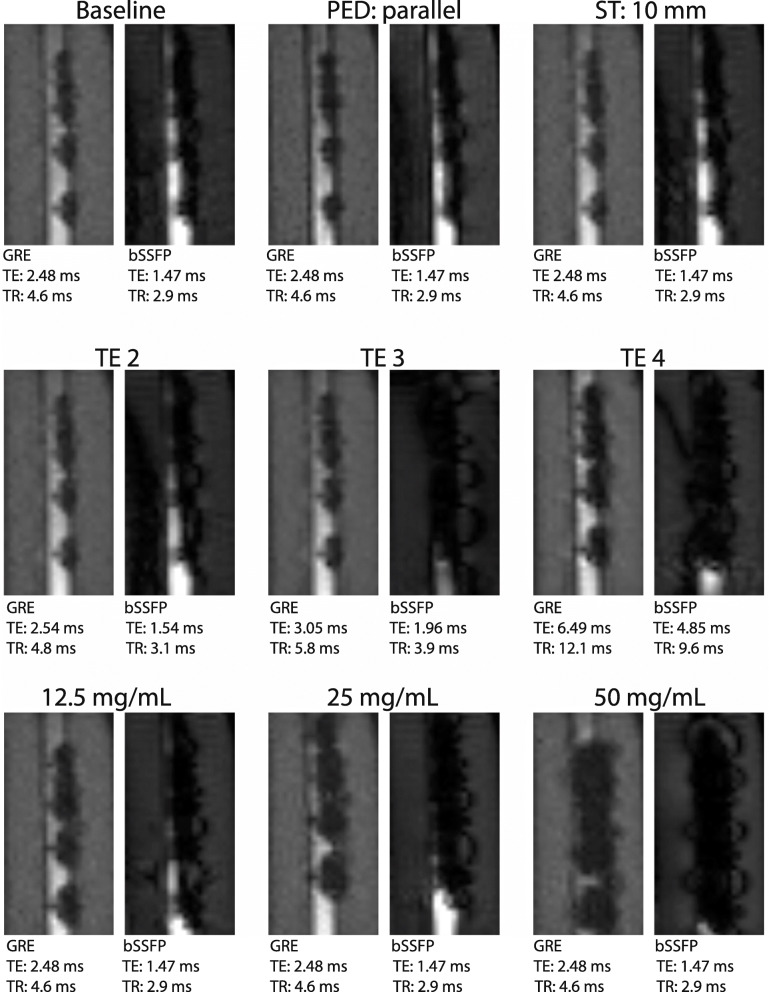
Pairs of gradient echo (GRE) and balanced steady-state free precession (bSSFP) images with iron(II,III)oxide (IO) marker artifacts for different combinations of technical parameters and IO concentrations. Baseline image: sagittal scan of phantom with inserted guidewire enhanced with 6.25 mg/mL IO markers (phase encoding direction [PED] perpendicular, slice thickness [ST] 5 mm). The other image pairs deviate from the baseline images with different PED, ST, echo time (TE), or IO concentrations. *TR* Repetition time

Per parameter setting, 25 images were available for image analysis. The bSSFP images with the highest TE (4.85 ms) showed severe banding artifacts extending within the area of interest, overlapping with the marker artifacts, and were therefore excluded from quantitative analysis. Exemplary passive marker artifacts for different imaging and marker parameters are shown in Fig. 3.

### Quantitative image analysis

**Fig. 4:**
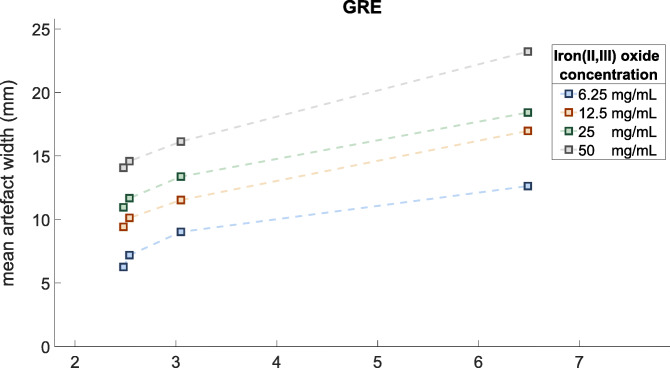
Graph displaying the cumulative effect of adjusting echo time and iron(II,III)oxide concentrations on mean artifact width for gradient echo images

**Fig. 5:**
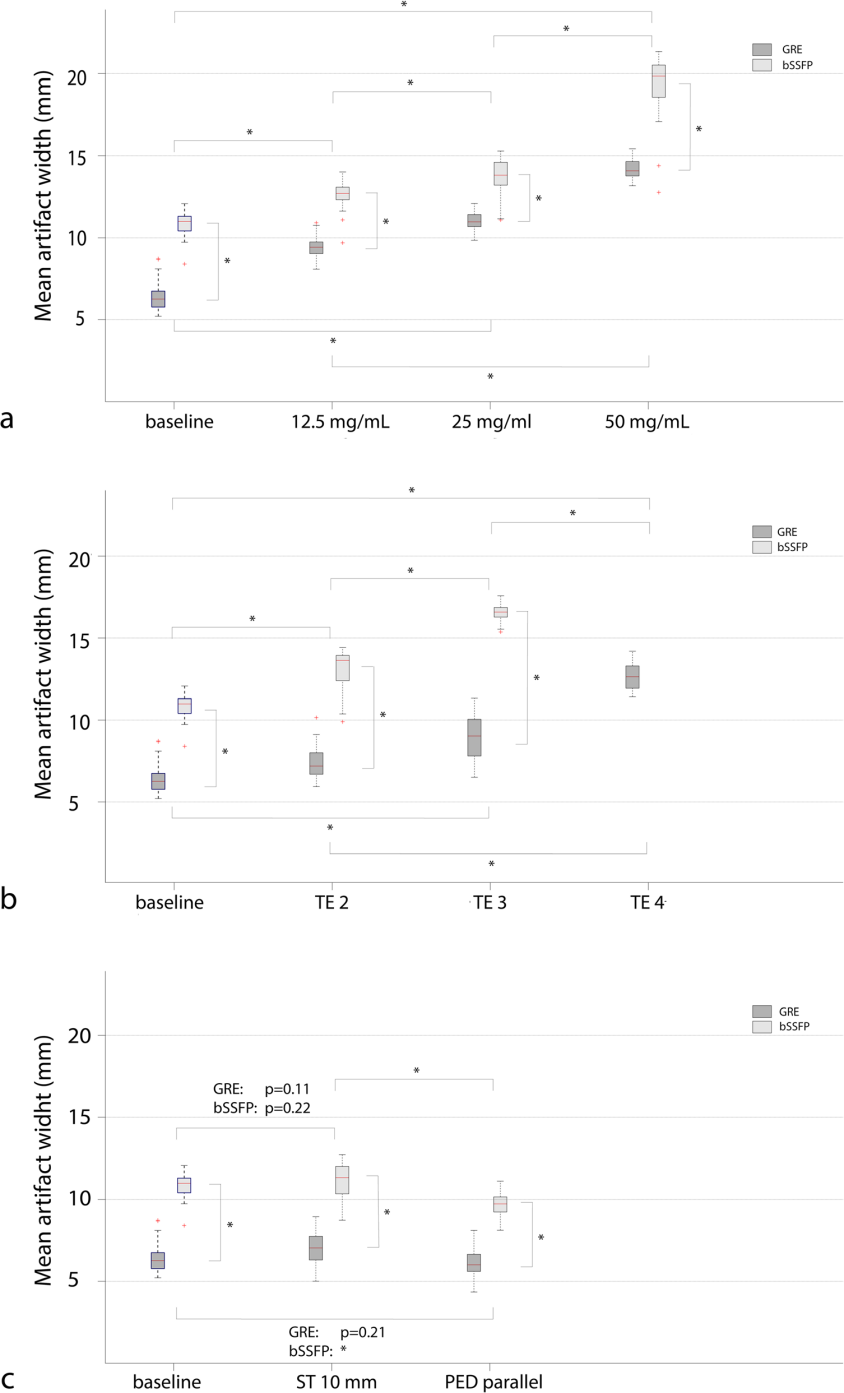
Boxplots of mean artifact width for the different parameters: iron(II,III)oxide concentration (**a**); echo times (TE) (**b**); slice thickness (ST) and phase encoding direction (PED) (**c**). *bSSFP* Balanced steady-state free precession, *GRE* Gradient echo. *p < 0.01

Mean artifact width was positively correlated with paramagnetic material concentration and TE for both sequences. Both TE and the IONP concentration contributed to the artifact size in a cumulative manner (Figs. 4 and 5). For the tested parameter range, mean artifact width in the GRE images ranged from 6.3 mm at lowest IONP concentration (6.25 mg/mL) and shortest TE (2.48 ms) to 23.2 mm at highest concentration (50 mg/mL) and longest TE (6.49 ms). The difference in artifact width between the various echo times and between marker concentrations was significant for each combination (Fig. 5). Effects of altering PED and ST had minimal impact on artifact width with deviations smaller than 1.5 mm. The influence of the evaluated parameters on CNRs is tabulated in Table 2. Blood-marker CNR was significantly higher for bSSFP images compared to GRE images (*p* < 0.001). Furthermore, the blood-artifact CNR showed a positive relationship with TE for the both sequences. The agar-artifact CNR was significantly lower (*p* < 0.001) compared to the blood-artifact CNR in all MRI images.

**Table 2:** Contrast-to-noise ratios for all evaluated parameters

Evaluated parameters	Median blood-artifact CNR (IQR)	***p***-value w.r.t baseline sequence	Median agar-artifact CNR (IQR)	***p***-value w.r.t baseline sequence
**GRE**				
Baseline sequence (a)	141.2 (129.4–150.0)		15.8 (11.2–19.9)	
Conc. 12.5 mg/mL	148.2 (133.1–155.3)	< 0.001	20.7 (16.6–23.6)	< 0.001
Conc. 25 mg/mL	148.5 (139.0–161.2)	< 0.001	25.7 (23.4–27.4)	< 0.001
Conc. 50 mg/mL	155.8 (141.3–162.1)	< 0.001	28.5 (27.4–29.7)	< 0.001
Sequence b	166.6 (156.8–175.3)	< 0.001	14.6 (3.6–20.1)	0.276
Sequence c	240.5 (224.4–248.5)	< 0.001	12.9 (7.2–25.4)	0.840
Sequence d	360.3 (349.9–366.2)	< 0.001	32.7 (27.9–38.0)	< 0.001
ST: 10 mm	133.8 (126.8–145.9)	0.042	10.5 (4.6–15.8)	0.014
PED: parallel	125.1 (116.9–137.6)	< 0.001	10.2 (4.7–57.5)	0.026
**bSSFP**				
Baseline sequence (e)	399.5 (389.5–406.3)		2.4 (-0.7–5.2)	
Conc. 12.5 mg/mL	387.3 (380.6–395.4)	0.003	37.6 (36.7–43.5)	< 0.001
Conc. 25 mg/mL	397.1 (393.4–402.6)	0.500	57.3 (54.2–62.1)	< 0.001
Conc. 50 mg/mL	478.9 (466.1–483.4)	< 0.001	53.3 (51.0–59.4)	< 0.001
Sequence f	572.6 (556.0–584.7)	< 0.001	17.1 (14.9–23.3)	< 0.001
Sequence g	766.2 (704.6–823.5)	< 0.001	59.6 (55.8–65.1)	< 0.001
Sequence h	NA.	NA	NA	NA
ST 10 mm	414.2 (402.5–423.6)	< 0.001	1.8 (-1.5–8.5)	0.443
PED parallel	264.0 (252.9–272.2)	< 0.001	2.3 (0.6–6.0)	0.800

*bSSFP* Balanced steady-state free precession, *CNR* Contrast-to-noise ratio, *Conc.* Concentration, *GRE* Gradient recalled echo, *NA* Not available, *IQR* Interquartile range, *PED* Phase encoding direction, *ST* Slice thickness, *TE* Echo time, *TR* Repetition time, *w.r.t.* With respect to. Baseline sequences (a, e) and modified sequences (b, c, d, f, g, and h) are described in Table 1

### Qualitative image analysis

**Fig. 6:**
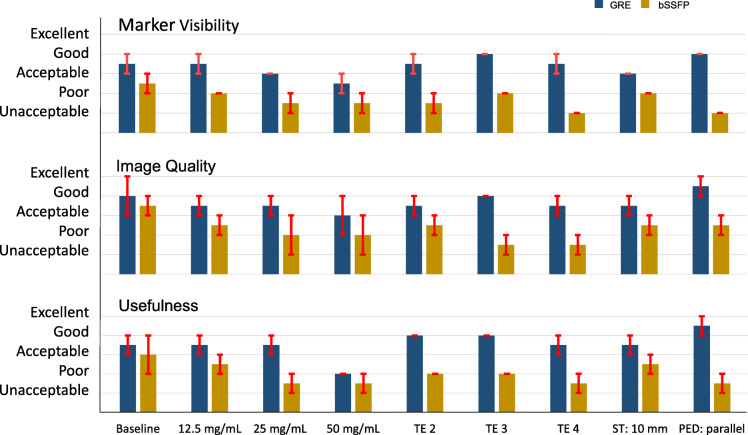
Median (blue and yellow bars) and individual observer (red bars) scores for marker visibility, image quality and usefulness of balanced steady-state free precession (bSSFP), and gradient echo (GRE) scans for the evaluated parameters: marker concentration, echo time (TE), slice thickness (ST), and phase encoding direction (PED)

The scores of the qualitative analysis are presented in Fig. 6. The GRE images were consistently better scored compared to the bSSFP images with mean scores of 3.3 *versus* 1.7 on marker visibility, 3.7 *versus* 2.3 on image quality and 3.6 *versus* 2.0 on usefulness, respectively. Except for the highest marker concentration, the scores for the GRE images were acceptable or good. The best performing bSSFP image was the baseline image. For the GRE sequence, images acquired with parallel PED performed best on all three topics.

### Automatic marker detection

**Fig. 7:**
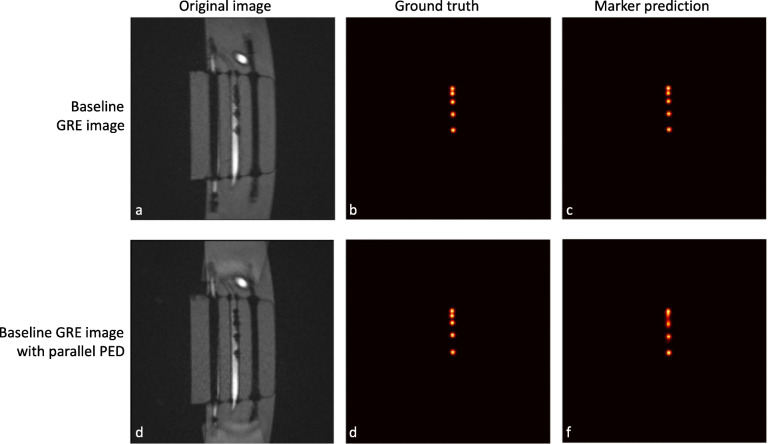
Original baseline (**a**) and parallel phase encoding direction (PED) (**d**) gradient recalled echo images with corresponding ground-truth annotations (**b**) and (**e**), respectively. Probability maps generated by the deep learning model of the original and parallel PED image (**c**, **f**), respectively. Note that the marker prediction of the top three markers is less distinct for the parallel PED image compared to the baseline image

The results of the automatic marker detection using the trained deep learning model are displayed in Table 3. Two marker location likelihood maps of the prediction by the model are shown in Fig. 7. The median number of markers that were correctly detected in the baseline, low TE (TE2 and TE3), and 10 mm slice thickness images was equal to the number of available markers in the images. The performance of the marker detection decreased for images with increasing concentration and TE, as well as for images with parallel PED.

**Table 3:** Number of mean correctly and wrongly detected markers for each threshold per dataset

Evaluated parameters of GRE images	Median correctly detected markers (IQR)	Median false positive detected markers (IQR)
Baseline sequence (a)	5.0 (5.0–5.0)	0.0 (0.0–0.0)
Conc. 12.5 mg/mL	4.3 (3.0–4.8)	0.0 (0.0–0.0)
Conc. 25 mg/mL	3.7 (3.5–3.8)	0.2 (0.1–0.2)
Conc. 50 mg/mL	2.4 (1.0–3.0)	0.0 (0.0–0.0)
Sequence b	5.0 (5.0–5.0)	0.0 (0.0–0.0)
Sequence c	5.0 (5.0–5.0)	0.0 (0.0–0.0)
Sequence d	3.9 (3.1–4.4)	0.0 (0.0–0.0)
ST 10 mm	5.0 (5.0–5.0)	0.0 (0.0–0.0)
PED parallel	4.0 (3.8–4.0)	0.0 (0.0–0.0)

*Conc.* Concentration, *GRE* Gradient echo, *IQR* Interquartile range, *PED* Phase encoding direction, *TE* Echo time, *TR* Repetition time, *ST* Slice thickness

^a^Maximum markers to be detected was 5. Baseline GRE sequence (a) and modified sequences (b, c, and d) are described in Table 1

## Discussion

This study shows that artifact visibility and size can be adjusted by changing TE or the amount of IONP for each marker, giving users and manufacturers the possibility to tailor visibility for a range of endovascular interventions. Artifact size and image quality varied for the different parameters and were favourable for the GRE images as compared to bSSFP. Furthermore, on the way towards time-efficient MRI-guided endovascular interventions, automatic marker detection was shown to be feasible but alterations in MRI or marker parameters should be taken into account to ensure reliable detection.

Based on our results, the GRE sequence is recommended for MRI-guidance of endovascular interventions since the images scored better in the image quality assessment. The bSSFP sequence is associated with a higher sensitivity for field inhomogeneities and therefore prone to banding and flow artifacts [16, 18, 21]. Although this can also be exploited, with larger artifacts facilitating easy identification (*e.g.*, in the aorta), this sequence appears less suitable for tracking moving markers in more distal arteries, despite the accompanying higher blood signal-to-noise ratio [23, 31].

Our study results under pulsatile conditions are in concordance with findings in static phantom studies [25, 32, 33]. A study of Bos et al. [25] showed that TE correlates with artifact size in agar phantoms. The same study simulated the effect of the magnetic susceptibility in relation to the signal intensity and reported a negative relation [25], corresponding with the larger artifacts for higher IONP concentrations in our study. The increase in artifact width when increasing ST, albeit a relatively minor effect, corresponds with literature [32]. Lastly, Lewin et al. [33] demonstrated that a parallel PED slightly decreased artifact width in needles for FISP sequences, whereas the FLASH sequence was not influenced [33]. Although the effects of the different parameters in experiments without flow point in the same direction, a direct comparison with our results is not possible. The pulsatile flow in our phantom caused motion of the wire, likely resulting in visible variations in marker artifacts in consecutive images. In this study, however, no evaluation on marker visibility and detectability between pulsatile and static flow was made since static experiments have been extensively described before.

In this study, it has been shown that deep learning-based automatic marker detection of the markers is feasible. The performance was comparable for small adjustments; however, larger increase in TE and marker concentration as well as adjusting the PED weakened the performance. Although other articles describe methods to automatically detect markers on endovascular devices with promising results [14, 34], we are unaware of literature describing the application of deep learning based models to detect the markers.

Several recommendations can be made for clinical use of passive marker enhanced devices for endovascular interventions at 3T MRI. Since artifact size and blood-artifact CNR are highly dependent on marker concentrations, the amount of paramagnetic material per marker requires critical evaluation in the production of vascular devices. The blood-artifact CNR increased for longer TE in bSSFP and GRE images, which is most likely related to the inflow of new blood in the imaging plane in combination with a higher TR. For the tested conditions and parameter range, a marker concentration lower or equal to 12.5 mg/mL appears desirable for navigating through medium-sized vessels. However, when navigating the guidewire through large vessels, larger artifacts may be useful to track the guidewire. Therefore, adjusting the artifact size by increasing the TE might be beneficial, albeit at the cost of lower signal-to-noise and temporal resolution. Furthermore, the images showed that markers with small intermarker distances tend to overlap in most scanning conditions. This also caused a decrease in the number of correctly detected markers by the model in some datasets. To prevent overlap of markers, distances between markers of at least 20 mm are recommended based on the images in this study. Finally, it has been shown that a basic deep learning model was able to detect the marker locations in GRE images. To improve the robustness of the model, training using MR images acquired with a wide range of varying parameters is recommended.

Main strength of this study is that marker visibility was evaluated in a clinically representative setting by using a vascularised phantom with tissue and blood mimicking MRI characteristics and realistic flow patterns. Furthermore, the evaluated sequences are generally accepted for MRI-guided endovascular interventions. In addition, this study combined both quantitative and qualitative analysis to enable investigation of the objective changes in the marker artifacts as well as the applicability and usefulness of the images according to expert evaluation.

This study is limited to a phantom setting, where measurements could be obtained under optimal conditions (*i.e.*, optimal coil positioning, no subject movement). Our findings need to be re-evaluated within *in vivo* data before clinical implementation is appropriate. Furthermore, in our study, clinical imaging protocols were used as baseline and adapted, whereby TE could not be changed independently from TR and bandwidth, yielding non-linear spacing between parameter values. Also, the difference between 1.5 T and 3 T main magnetic field has not been investigated in the present study, but could have provided valuable insight in the generalizability of the findings in this study, since 1.5 T systems are generally used in cardiac interventions [3, 16]. Furthermore, the area and volume of the marker artifacts were not evaluated in three-dimensions due to the use of single two-dimensional slices, which may have provided additional information.

The acquired knowledge in the current study can be used to optimise visibility of passive marker endovascular devices and aid automatic detection within human subjects. Future studies, where the marker detection algorithm is trained and tested within patient data, are required as a crucial next step towards automation of MRI scanning during vascular interventions [35]. Ultimately, this may enable an artificial intelligence-based marker detection system which could analyse the image stream in real-time, continuously tracking the guidewire position and updating the MR image planes to enable real-time device tracking with optimised imaging scan planes  [36, 37].

In conclusion, this study has demonstrated that MRI conditional guidewires with passive paramagnetic markers provide sufficient visibility for MRI guidance at 3 T in a pulsatile flow phantom. A large range of artifact sizes can be achieved, which was predominantly dependent of TE and IONP concentration. Lastly, automatic markers detection was feasible; however, adjusting TE, PED, and IONP concentration influenced marker detection performance. Depending on the clinical application, the sequence type and imaging parameters, predominantly TE, need to be optimised to tailor device visibility and detectability during MRI-guided endovascular interventions.

## Data Availability

Data are available from the corresponding author based on reasonable request.

## Abbreviations

bSSFP: Balanced steady-state free precession
CNR: Contrast-to-noise ratio
GRE: Gradient recalled echo
IONP: Iron(II,III) oxide nanoparticles
PED: Phase encoding direction
ST: Slice thickness
TE: Echo time
TR: Repetition time
